# Higher heavy metal contamination indoors than outdoors during COVID-19 in Mexico City

**DOI:** 10.1007/s11356-024-32085-8

**Published:** 2024-02-07

**Authors:** Anahí Aguilera, Ángeles Gallegos, Víctor Luna, Luciano Hernández, Margarita Gutiérrez, Daniel Amaro, Avto Goguitchaichvili, Patricia Quintana, Francisco Bautista

**Affiliations:** 1 Centro de Investigaciones en Geografía Ambiental, Laboratorio Universitario de Geofísica Ambiental, Universidad Nacional Autónoma de México, Antigua Carretera a Pátzcuaro No. 8701, Col. Ex-Hacienda de San José de La Huerta, C.P, 58190 Morelia, Michoacan Mexico; 2 Facultad de Química, Universidad Nacional Autónoma de México, Ciudad de Mexico, Mexico; 3 Instituto de Geofísica, Laboratorio Universitario de Geofísica Ambiental, Universidad Nacional Autónoma de México, Antigua Carretera a Pátzcuaro No. 8701, Col. Ex-Hacienda de San José de La Huerta, C.P, 58190 Morelia, Michoacan Mexico; 4 Departamento de Física Aplicada, Centro de Investigación y de Estudios Avanzados. Carr. Mérida - Progreso, Loma Bonita, 97205 Merida, Yucatan Mexico; 5 Colegio de Postgraduados, Periférico Carlos A. Molina S/N Km. 3, Periférico Carlos A Molina SN, Ranchería Río Seco y Montaña, 86500 Heroica Cardenas, Tabasco Mexico

**Keywords:** Urban dust, Road dust, Health risk, Pollution, Lead, Zinc, Hazard index

## Abstract

People spend most of their time indoors, especially during the coronavirus disease. Prolonged exposure to heavy metal-contaminated dust can be harmful to human health. The objectives of this study were to identify the contamination level in outdoor and indoor dust, compare contamination in both environments, and assess the human health risk. Two-hundred thirty-nine samples of dust were taken by Mexico City citizens in 38 homes on the weekends of May 2020. Heavy metal concentrations were measured through XRF. The contamination level was set using the contamination factor with a local and global background value, mixed linear models were used to identify indoor and outdoor differences, and USEPA human health risk methodology was used. Pb, Zn, and Cu had the highest contamination levels, followed by Sr and Mn, using both the local and global background values. The Pb, Zn, and Cu contamination was greater indoors, while higher Mn, Sr, and Fe were detected outdoors. According to the outdoor/indoor ratios, the main sources of Ca, Pb, Zn, and Cu must be indoors, while the main sources of Fe, Mn, Sr, Y, and Ti are outdoors. A human health risk was not detected, as the hazard index was lower than one. However, ailments can be developed due to exposure to Pb, Mn, and Fe in children (hazard index > 0.1). A higher risk due to Pb exposition was found indoors. Indoor environments in Mexico City were more contaminated by heavy metals and represented a higher risk to human health than outdoors during the pandemic isolation.

## Introduction

Currently, 55% of the world’s population lives in urban areas, and it is estimated that by 2050 this figure will break 68% (Hassanein et al. 2019). The exposure of city dwellers to urban dust pollutants is a very worrying issue. Dust is a complex mixture of organic and inorganic components that come from various sources, both natural and anthropogenic, such as fires, industrial and vehicle combustion processes, and the wear and tear of construction materials and soils. Dust is both a medium and a vehicle for metals and other contaminants because it can transport contaminants by resuspension into the air and through rainwater runoff (Aguilera et al. 2021a).

Outdoor dust can enter indoor environments through air exchange or adhering to the soles of shoes. In addition, the materials of the houses and the activities of the residents can generate indoor dust (Dingle et al. 2021), for example, the daily burning of fuel in the kitchen, air conditioning, paints, cigarette lighting, and the building age (Dingle et al. 2021). The exposure time to indoor dust in homes is longer than outdoors. Children are at greater risk than adults since they have more contact with surfaces, as well as the habit of putting their hands and objects in their mouths (Jadoon et al. 2018). Previous studies have shown that oral ingestion is the main route of exposure in homes (Zhou et al. 2022). Ingested dust reaches the gastrointestinal tract, where the heavy metals are partially dissolved and transported by the circulatory system to eventually accumulate in target tissues and organs of the human body (Rasmussen et al. 2018).

Urban dust represents a risk to human health due to two main reasons: (1) particle size, smaller particles, represents more harm to health (Chen et al. 2016) and (2) the dust composition. Heavy metals are among the adhered or absorbed contaminants in the structure of the dust particles; heavy metals are persistent and bio-accumulative. Dust particles enter the human body through ingestion, inhalation, and dermal contact. The adverse effects on human health will depend on factors such as the element type, its shape, metabolic route, exposure time, and the susceptibility of each person. Non-essential elements such as As, Cd, Cr, Hg, and Pb are lethal even in low concentrations since they accumulate in tissues (Tchounwou et al. 2012), affect the central nervous system, can be deposited in the circulatory system and disrupt the normal functioning of internal organs, are cofactors of cardiovascular and respiratory diseases, and can cause DNA damage, such as mutagenic, teratogenic, and carcinogenic effects (Safiur Rahman et al. 2019; Tchounwou et al. 2012).

In cities with an economic sector based on trade and transport, and an increase in their population, there are various fixed and mobile sources of heavy metals (or dust contaminated with heavy metals). Mexico City is one of the largest and most populous cities in the world, with high pollution levels (Aguilera et al. 2021b; Delgado et al. 2019). In 2018, this city was the fourth largest agglomeration in the world and the first in the American continent, behind Tokyo, Delhi, and Shanghai (SUN 2018). It has around 23 and a half million inhabitants, 40,000 small- and medium-sized industries, and 9.5 million vehicles (Morales et al. 2020).

Although it is known that in Mexico City there is high contamination by heavy metals in outdoor dust, the situation of indoor dust is less known; higher PM concentrations have been reported indoors than outdoors (Reynoso-Cruces et al. 2023). Indoor dust is an important source of exposure because the population spends most of the time in these environments, especially during the COVID-19 pandemic, when people were instructed to stay indoors as much as possible. Therefore, the main contribution of this research was to evaluate the contamination by heavy metals in outdoor and indoor dust of the houses of Mexico City, seeking to prove if the outdoor dust is the main source of heavy metals in the interior or if indoor sources have an important contribution; indirectly, this question was also addressed: the COVID-19 isolation changed polluting patterns by decreasing outdoor activities but increasing indoor ones? In addition, the human health risk was assessed using the USEPA methodology.

## Methodology

### Sampling and geochemical analysis

During the confinement of the COVID-19 pandemic, the population of Mexico City was summoned to a participatory sampling of indoor and outdoor dust from their homes. The invitation was addressed mainly to the National Autonomous University of Mexico students and their families, although anyone could participate. The participants received training on sample collection through videos, and a mobile app was developed for sample information registration, such as the location and characteristics of the dwelling, such as the floor types because this information was included in the mixed linear models. To obtain the outdoor dust sample, 1 m^2^ of sidewalk surface area was swept, and the entire house was swept to collect the indoor dust. To comply with the sampling methodology, participants uploaded photographs of the sampling area.

**Fig. 1:**
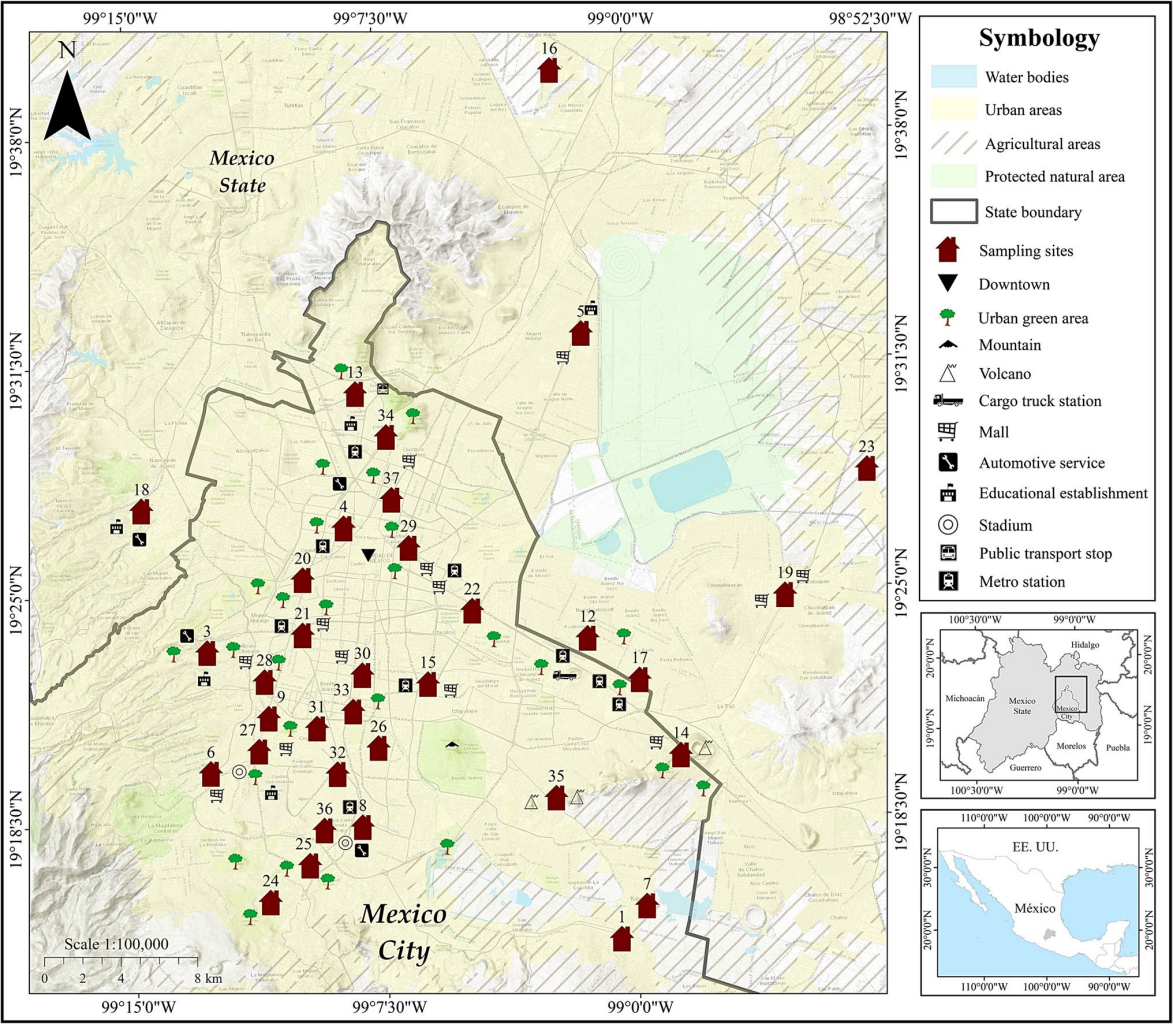
Geographical location of sampled sites

Samples were collected on weekends in May 2020; before the rainy season, they were stored in resealable polyethylene bags and labeled. In total, 239 dust samples were obtained (140 outdoors and 99 indoors) and collected from 38 houses along Mexico City (Fig. 1). In the laboratory, the samples were left to dry in the shade to avoid oxidation of the minerals present. Subsequently, they were sieved with a 60 mesh (250 µm).

Three grams of dust was placed in appropriate XRF sample holders, consisting of a Teflon cup, with a bottom opening covered with a thin 3.6-µm-thick Mylar film (polyester). Metal concentrations were measured with a Genius 7000 portable XRF spectrometer from Skyray Instruments: an X-ray tube of 50 kV with a large-area beryllium-window silicon shunt detector. The measurements were made in triplicate, with an integration time of 60 s; then, the average and percentage or relative standard deviation were obtained, which were used to evaluate the precision of the measurements. A variation of less than 20% was considered acceptable. Elements with variations greater than 20% were discarded from the analysis. To evaluate the accuracy of the measurements, the IGLs-1 standard was used, which is composed of lateritic soil, rich in halloysite, hematite, maghemite, goethite, and quartz (Lozano and Bernal 2005).

### Mineral analysis

The four most polluted samples were selected to analyze urban dust minerals, two indoors and two outdoors. The samples were submitted to XRD after the following pre-treatments: air-dried, ethylene glycol solvated, and heated at 550 °C for 1 h.

To guarantee the quality of the analysis, we use XRD patterns obtained with a Siemens D-5000 diffractometer, with Cu Kα radiation (*l* = 1.5418 Å) operated at 34 kV and 25 mA, from 2° to 50° (2q), with steps of 0.02° 2q at 3 s/step. Crystalline phases were identified using the ICDD-PDF (2000) and Dana’s Mineralogy (Gaines et al. 1997).

### Contamination level indoors and outdoors

The contamination factor (CF) and the pollution load index (*PLI*) were used to assess the level of outdoor and indoor heavy metal contamination. The CF was calculated with the following formula:

1 $$FC=Cn/Bn$$

where *Cn* is the concentration of each heavy metal and *Bn* is the corresponding background value. In this research, two background values were used, a local one that was the first decile of the frequency distribution of each metal indoors and outdoors and a global one that was the one reported for soils worldwide (Kabata-Pendias 2011). A CF less than 1 indicates *insignificant contamination*, 1–3 indicates *moderate contamination*, 3–6 represents *considerable contamination*, and more than 6 denotes *high contamination*.

The *PLI* is the geometric average of the CFs of the five most contaminated metals. It was calculated using the following equation:

2 $$PLI=\sqrt[n]{CF1*CF2*\dots *CFn}$$

A *PLI* greater than 1 indicates heavy metal contamination. This index was developed to assess contamination in sediments (Tomlinson et al. 1980). Urban dust is a form of sediment from atmospheric particles that settle on impervious surfaces of streets and houses.

### Comparison of indoor and outdoor heavy metal concentrations

To compare the concentrations of heavy metals outdoors and indoors, a mixed linear model was used, and the type of collection (outdoors or indoors) was introduced as a fixed effect in the model. The collection date and the fraction in which heavy metal concentrations were measured (595 and 250 μm) were also entered as fixed effects, only to control for their contribution to model variation. The variation that could be introduced by the location of the houses was counted as a random effect because the samples taken in the same location (house) were not independent among themselves.

The mixed linear model was fitted with the lmer function of the lme4 package from the R project software, version 4.2.0 (2022–04-22 ucrt) “Vigorous Calisthenics.” Subsequently, it was tested that the assumptions of the model were met, i.e., normality and homoscedasticity of the residuals, as well as normality of the random effects, through graphs. When these assumptions were not met, a generalized linear mixed model with gamma distribution (glmer function from the lme4 package) or a mixed linear model with logarithmic transformation was used. Finally, the statistical inference on the effects of the type of collection, the date, and the fraction was carried out through hypothesis tests with the ANOVA type II function of the lme4 package.

Mixed linear models were also adjusted to the type of surface on which the indoor dust sample was collected, following the methodology described in the previous paragraphs, to identify if the floor material of the house influenced the concentrations of heavy metals.

Subsequently, outdoor/indoor (O/I) ratios were used to assess the impact of outdoor dust on indoor environments. An O/I greater than 1 refers to the fact that the main sources of heavy metals are outside; on the contrary, an O/I less than 1 indicates that the sources are mainly inside homes (Zhou et al. 2022). Together with the O/I analysis, the Pearson and Spearman correlations were reviewed to investigate whether there was an association between the outdoor and indoor concentrations of each heavy metal. A high correlation coefficient suggests that the sources of the metals may be the same.

### Human health risk assessment

To estimate the human health risk of heavy metals present in outdoor and indoor dust, the USEPA methodology was used. In the first instance, estimated daily intakes were calculated for each route of exposure: ingestion (${EDI}_{ing}$), inhalation (${EDI}_{inh}$) and dermal contact (${EDI}_{dermal}$) (Eqs. 3–5) as well as Lifetime Average Daily Dose (*LADD*) to estimate the carcinogenic risk (Eq. 6).

3 $${EDI}_{ing}=\frac{C*IngR*EF*ED*CF}{BW*AT}$$

4 $${EDI}_{inh}=\frac{C*InhR*EF*ED}{PEF*BW*AT}$$

5 $${EDI}_{dermal}=\frac{C*SA*AF*ABS*EF*ED*CF}{BW*AT}$$

6 $$LADD=\frac{C}{PEF\times {AT}_{can}}\times \left(\frac{{CR}_{kids}\times {EF}_{kids}\times {ED}_{kids}}{{BW}_{kids}}+\frac{{CR}_{adults}\times {EF}_{adults}\times {ED}_{adults}}{{BW}_{adults}}\right)$$

*CR* is the contact or absorption rate.

7 $${HQ}_{ing/inh/derm}=\frac{{EDI}_{ing/inh/derm}}{RfD}$$

In this study, we calculated the *EDI* for each sampling point, both outdoors and indoors. The use of local parameters could improve the reliability of the estimates; however, exposure factors have not been estimated for any Mexican city, so those of reference populations were used (Table 1).

**Table 1:** Exposure factors to estimate the risk to human health

Factor	Definition and units	Value		Reference
		Child	Adult	
*IngR*	Ingestion rate (mg day^−1^)	200	100	USEPA (2011)
*InhR*	Inhalation rate (m^3^ day^−1^)	7.63	12.8	Li et al. (2001)
*PEF*	Particle emission factor	1.36E + 09	1.36E + 09	USEPA (2001)
*SA*	Surface of exposed skin area (cm^2^)	2800	5700	USEPA (2001)
*ABS*	Dermal absorption factor	0.001	0.001	Ali et al. (2017)
*AF*	Skin adherence factor (mg cm^−2^)	0.2	0.07	USEPA (2001)
*ED*	Duration of exposure (years)	6	24	USEPA (2001)
*EF*	Frequency of exposure (days yr^−1^)	350	350	Zheng et al. (2010)
*AT*	Average time non-carcinogens (days)	ED × 365	ED × 365	USEPA (1989)
*At*_*can*_	Average time for carcinogens (days)	70 × 365	70 × 365	USEPA (1989)
*BW*	Body weight (kg)	15	70	Kurt-Karakus (2012) and Mohmand et al. (2015)
*C*	Heavy metal concentration (mg kg^−1^)	Measured at each sampling site		
*CF*	Conversion factor (kg mg^−1^)	1 × 10^−6^		Li et al. (2001)

Hazard quotients for each exposure route: ingestion, inhalation, and dermal contact (${HQ}_{ing/inh/derm}$), were obtained by dividing the $EDI$ by the corresponding reference doses ($RfD$), as shown in Eq. 7. Reference doses (Table 2) were collected in a previous literature review by ourselves as the most cited values reported in the scientific articles reviewed (Aguilera et al. 2021a, 2021b).

**Table 2:** Reference doses (*RfD*) and slope factors (*CSF*) for each route of exposure

	Oral *RfD*	Inhalation *RfD*	Dermal *RfD*	Oral *CSF*	Dermal *CSF*	Inhalation *CSF*
Cu	4.00E − 02	4.02E − 02	1.20E − 02			
Fe	8.40E + 00	2.20E − 04	7.00E − 02			
Mn	4.60E − 02	1.43E − 05	1.85E − 03			
Pb	3.50E − 03	3.52E − 03	5.25E − 04	0.0085		4.20E − 02
Zn	3.00E − 01	3.00E − 01	6.00E − 02			

This values were collected in a previous literature review by ourselves (Aguilera et al. 2021a, 2021b)

An accepted or tolerable risk ranges from 1E − 06 to 1E − 04 (USEPA 2001). These values indicate that one additional case in a population of 1,000,000 and 10,000 people, respectively, is acceptable

The non-carcinogenic risk index (HI) is made up of the sum of the $HQ$ for the three exposure routes. If the HI is greater than 1, it means that there is a risk to human health; if it is less than 1, there is no risk to the health of the population (USEPA 2001).

The lifetime risk of developing cancer (*ILCR*) was only calculated for Pb, as this is the only carcinogenic metal we studied, using Eq. 8, for both exposure routes (Table 2). Therefore, the *ILCR* represents the sum of both oral and inhalation routes.

8 $$ILCR=LADD\times CSF$$

## Results and discussion

All metals, outdoors, had an asymmetric frequency distribution. The same was held indoors, except for Mn, which did have a normal or symmetrical distribution, according to the Shapiro normality test. An asymmetric frequency distribution is very common in environmental studies on heavy metal concentrations since generally there are few sites with very high concentrations (Guvenç et al. 2003). The metal concentrations decreased in the order Ca > Fe > Ti > Mn > Zn > Sr > Cu > Pb > Y. In a previous study in Mexico City, with only outdoor dust samples, we found the same order in the concentrations of the heavy metals analyzed, with the exception that the medians of Mn and Zn were inverted; at that time there were higher concentrations of Zn than of Mn and now it was the opposite (Aguilera et al. 2021b). Possible reasons for the change in the order of Mn and Zn are an increase in the Mn emissions or decrease in the Zn ones in the city.

The highest coefficients of variation were those for Cu, Pb, and Zn, both outdoors and indoors (Table 3). This is an indication that these metals have an anthropogenic origin. Generally, these metals have been associated with vehicular traffic; however, in Mexico City, the presence of Pb in the air is related to the resuspension of polluting soil, industries, and some paints and pigments (Instituto Nacional de Ecología y Cambio Climático 2019).

**Table 3:** Descriptive statistics for the heavy metal concentrations (mg kg^−1^) in outdoor and indoor dust in Mexico City

*metal*	*min*	*max*	*Median*	*mean*	*std.dev*	*coef.var*	*D1*	*global*
Outdoors (*n* = 140)								
Ca	43,325.0	157,286.3	64,613.4	69,048.6	19,665.6	0.3	51,333.1	
Fe	16,605.8	86,463.0	31,839.5	32,619.2	7244.9	0.2	26,791.0	
Ti	2209.6	13,078.5	5187.4	5317.3	1207.9	0.2	4602.5	70.4
Mn	302.2	1653.3	707.7	729.6	193.6	0.3	558.9	488.0
Zn	95.5	1704.1	500.9	569.4	310.1	0.5	232.5	70.0
Cu	14.1	939.3	95.3	140.8	140.2	1.0	43.7	38.9
Pb	14.3	582.7	45.4	74.7	86.9	1.2	23.4	27.0
Sr	213.3	630.9	457.2	456.5	54.3	0.1	397.5	175.0
Y	24.6	38.7	32.7	32.8	1.9	0.1	30.8	23.0
Indoors (*n* = 99)								
Ca	29,126.9	168,783.7	64,874.6	75,410.6	30,956.2	0.4	45,762.4	
Fe	6228.6	69,240.9	27,267.2	28,482.5	10,463.6	0.4	16,624.7	
Ti	1930.7	15,584.5	5028.0	5276.7	1977.3	0.4	3388.6	70.4
Mn	119.4	1117.6	599.0	572.4	203.8	0.4	296.1	488.0
Zn	180.2	4589.0	855.2	1078.1	850.9	0.8	309.7	70.0
Cu	27.6	1434.9	129.3	217.5	252.9	1.2	52.1	38.9
Pb	17.0	373.4	76.9	98.1	81.2	0.8	22.9	27.0
Sr	70.9	696.5	405.9	377.9	116.8	0.3	190.9	175.0
Y	12.3	41.6	31.3	30.2	5.2	0.2	23.5	23.0

*min* minimum, *max* maximum, *std.dev* standard deviation, *coef.var* coefficient of variation, *D1* first decile (local background value), *global*: background value Kabata-Pendias (2011)

There is a great concern due to Pb contamination worldwide and, in particular, in Mexican cities, since it is the metal with the highest concentrations in outdoor dust and represents an ecological and children’s health risk (Aguilera et al. 2022). Pb concentrations in this study are lower than those found in a previous study in Mexico City (Morales et al. 2020). In 2016 the average concentration of Pb was 122 mg/kg, while in 2020 (the sampling year for this study) the average concentration was 74.7 mg/kg. In both cases, the Pb concentrations were measured with the same equipment, particle size, and the same methodology. In both studies, the entire city was analyzed; however, the sampling points were not exactly the same and variations in metal concentrations in street dust are very high even at close distances. These variations could be a reason for the decrease in Pb concentrations over these 4 years; another possible explanation is the reduction in heavy metal emissions due to industrial activity cessation and traffic reduction during the COVID-19 pandemic.

### Contamination level indoors and outdoors

The highest contamination factors were those for Pb, Zn, and Cu, followed by Sr and Mn, using both local and global background values (Table 3). Those metals were used to calculate the *PLI* to summarize the level of contamination by heavy metals in outdoor and indoor dust of homes in Mexico City. Ninety-six percent of the outdoor dust samples and 100% of the indoor dust samples were contaminated (*PLI* > 1) when the local background values were used, while all samples (outdoor and indoor) were polluted when the global background values were used.

**Fig. 2:**
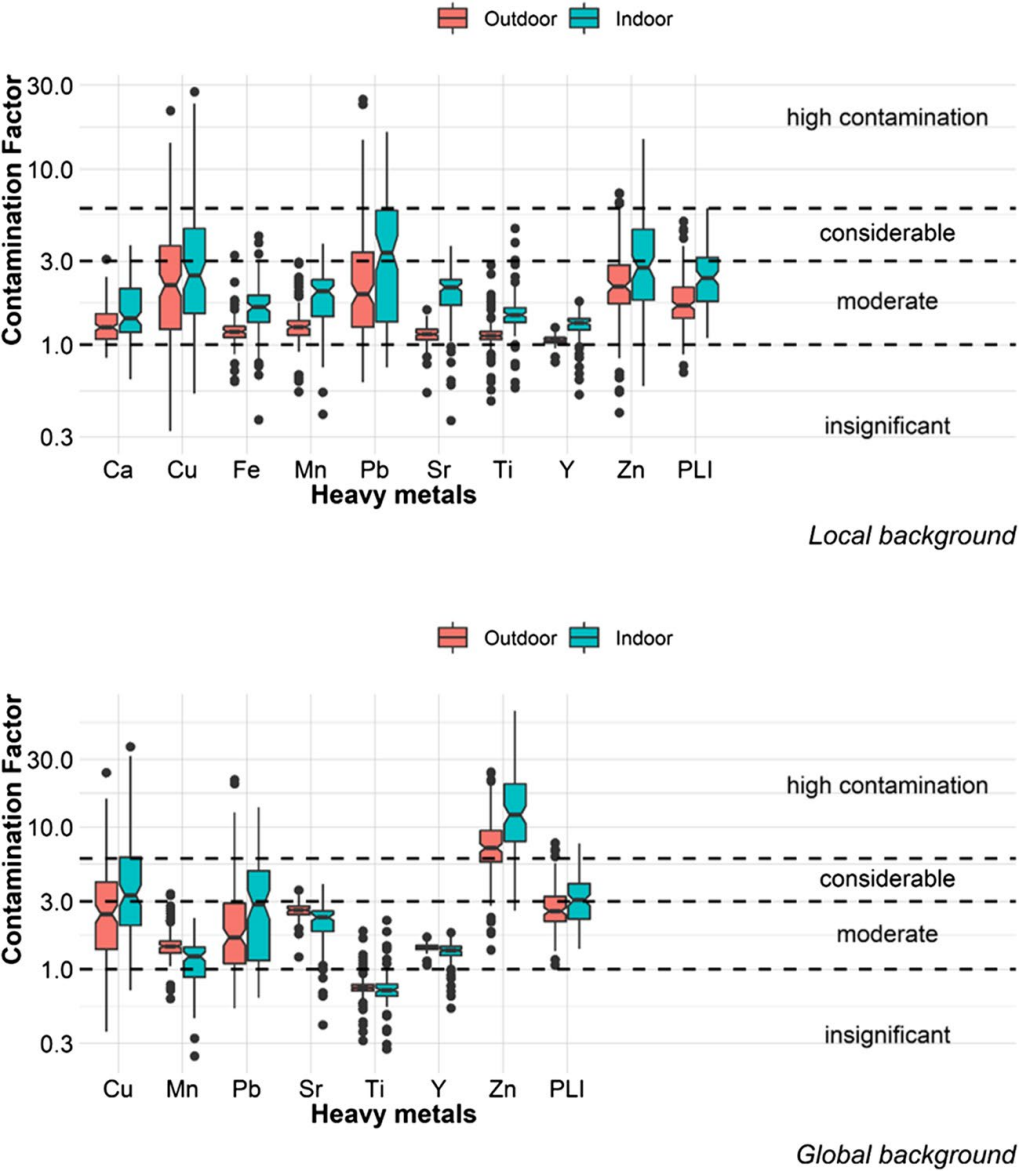
The highest contamination factors were those for Pb, Zn, and Cu, followed by Sr and Mn, using both local (top image) and global background values (bottom image). When data are visually compared, the Pb, Zn, and Cu contamination seem to be greater indoors

Contamination was higher when the global background value was used because the local background values were higher than the global ones (Table 3). This indicates that in Mexico City there is high contamination naturally; in addition, human activities had increased the contamination. It is important to highlight that in the case of urban dust, it is difficult to establish background values since, by definition, dust is a heterogeneous mixture of particles that come from both natural and anthropogenic sources. Cities are human-made places; therefore, it is difficult to establish values of naturally occurring metals within urban areas. However, the importance of using both background values, a local one and a global one, lies in the fact that through their comparison we can find out if there are high or low “natural” concentrations in a particular city concerning the global background value (Fig. 2).

The dust of Mexico City, compared to that of other cities in the country, such as Mérida and Morelia (Aguilera et al. 2022), has high background values. This may be due, on the one hand, to its volcanic origin and, on the other hand, to the fact that it is an ancient megacity, almost 700 years old. During all these years, it has been accumulating heavy metals as a result of human activities.

Among the analyzed metals, Zn stood out, since it presented high contamination with the global background value, for around 75% of the outdoor samples and more than 75% of the indoor ones (Fig. 2). However, it is important to mention that the analytical concentration measurement technique (portable XRF) seems to yield higher Zn values than other techniques such as ICP-OES. In a previous study from Mexico City, carried out by Morales et al. (2020), they compared the Zn concentrations measured with portable XRF and with ICP-OES and found an *R*^2^ of 0.9 in the linear regression; however, the measured concentrations with portable XRF were almost double those obtained with ICP-OES. This XRF equipment was the same one used to measure the concentrations of the metals in the present study. The average Zn in street dust from 2016 (sampling year in Morales et al. 2020) to 2020 sampled for this research has increased by 13%, 495.5 and 569.4 mg/kg, respectively.

With the local background value, contamination was higher inside homes for all metals; with the global background value, the contamination also turned out to be higher inside the homes for Cu, Pb, and Zn, while it was higher outside for Mn, Sr, and Y. The contamination seemed similar for Ti, outside and inside the houses. These differences are because the local background value was estimated separately for outdoors and indoors, so the values differ in each case. This means that indoor local background values were smaller than the outdoors ones, so less pollution was naturally expected inside the houses; however, high heavy metal concentrations were found respect to the background values. Global background values were the same outdoors and indoors, so we can compare the absolute concentrations through this figure; statistical analyses are represented in this figure.

### Comparison of indoor and outdoor heavy metal concentrations

The Pb, Zn, and Cu contamination were significantly greater indoors, while higher Mn, Sr, and Fe were detected outdoors. Initially, we thought that we would find higher concentrations of heavy metal outdoors since cars (Safiur Rahman et al. 2019) and industries (Aguilera et al. 2019) are reported to be some of the main sources of heavy metals in cities. However, we found the opposite: higher concentrations indoors than outdoors, specifically for those metals reported to have an anthropogenic origin in urban areas, such as Cu, Pb, and Zn. We consider that there may be three possible explanations for the higher Cu, Pb, and Zn content indoors, which are not exclusive but can occur at the same time: (1) there are important sources of these metals inside the homes; (2) there is a greater accumulation of metals indoors, especially if ventilation is poor; and (3) due to the decrease in outdoor activities because of the COVID-19 confinement, outdoor emissions decreased while indoors ones increased.

In a study conducted in Alberta, Canada, concentrations of Cu, Zn, and Pb indoors were 3 to 6 times higher than outdoors, concluding that there are indoor sources of these elements. The authors observed that the Pb concentrations decreased according to the age of the houses, going from 119 mg/kg in the oldest houses to 62 mg/kg in the newest ones, without significant differences. The highest anomalous concentrations were associated with the floor surface type; on cement or unfinished floors the highest concentrations of Pb were found, with significant differences. For other elements present in cement (Cu, Zn, Ni, Cr) higher concentrations were also found on cement surfaces, suggesting that cement dust is an important source of heavy metals, including Pb (Dingle et al. 2021).

In Mexico City, the participants in our study only recorded floors made of cement, mosaic, wood, or some of their combinations. The mixed linear models did not show significant differences between these types of surfaces. This is not consistent with what was reported by Dingle et al. (2021); although the floor materials are different between the two studies, we expected to find differences between cement and mosaic floors. However, it is important to mention that all the houses in our study and, in general, all the houses in Mexico City are made of cement and bricks, so the indoor dust must have particles of cement wear, regardless of the type of flooring material.

**Fig. 3:**
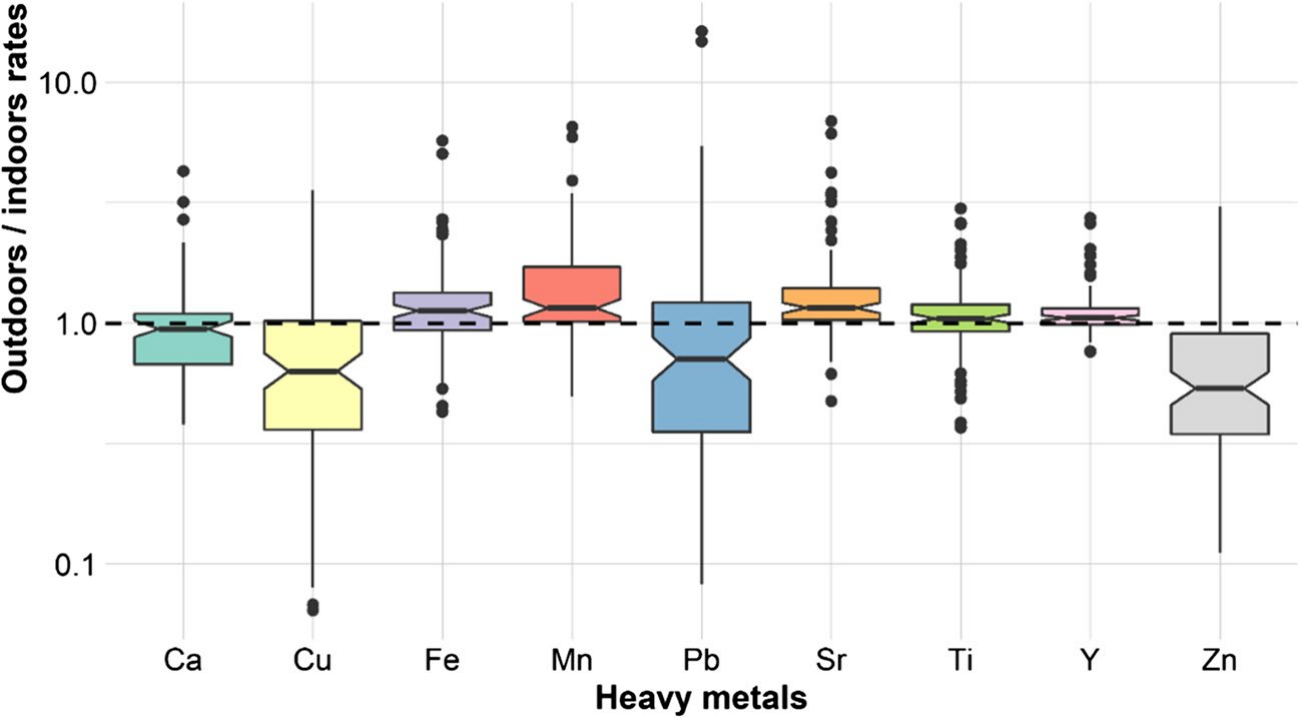
Outdoor/indoor rates for Cu, Pb, Zn, and Ca were lower than 1, indicating indoor sources, while O/I for Fe, Mn, Sr, and Ti were higher than 1, indicating outdoor sources

The outdoor-indoor ratio of heavy metal concentrations (O/I) may reflect the importance of outdoor sources versus indoor ones. The O/I ratio value is considered an appropriate indicator of the relative intensity of indoor versus outdoor sources (Zhou et al. 2022). The O/I ratios of Cu, Pb, Zn, and Ca, for most of the cases, were less than 1; this indicates that the main sources of these metals must be indoors. On the contrary, in most cases, the O/I ratios for Fe, Mn, Sr, Y, and Ti were greater than 1, which suggests that the main sources of these metals are outdoors. However, for at least 25% of the data for all metals, the O/I ratios were found to be in the opposite category; that is, for those with mainly indoor sources, 25% of the observations had outdoor sources and vice versa (Fig. 3). Indoor heavy metal concentrations decreased as the O/I ratio increased, reinforcing the idea that indoor sources provide approximately 75% of the Ca, Cu, Pb, and Zn, and 25% of the rest of the elements. In contrast, O/I ratios greater than 1 for Ca, Cu, Pb, and Zn were not related to higher concentrations of each element outdoors; this is probably a random effect.

**Fig. 4:**
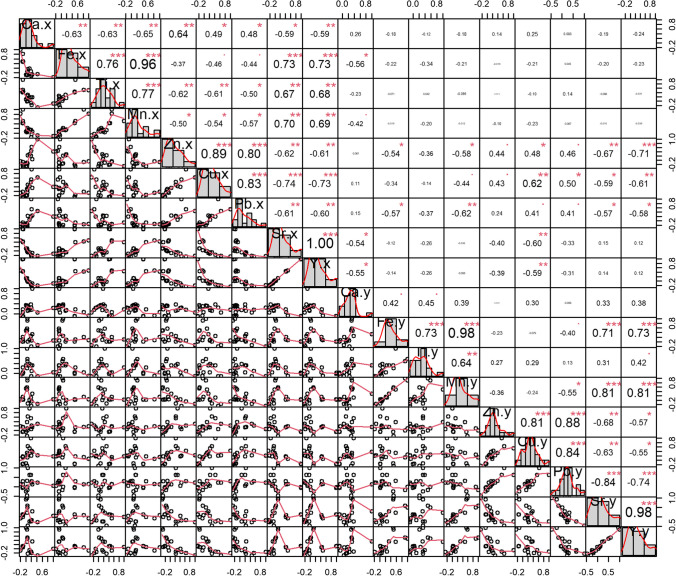
Spearman correlations coefficients between outdoor (Ca.x, Fe.x, Ti.x Mn.x, Zn.x, Cu.x, Pb.x, Sr.x, Y.x) and indoor (Ca.y, Fe.y, Ti.y, Mn.y, Zn.y, Cu.y, Pb.y, Sr.y, Y.y) heavy metal concentrations

We found a strong positive correlation between outdoor and indoor Cu concentrations (*r* = 0.6); for the rest of the metals, the Pearson and Spearman correlation coefficients were less than 0.5 (Fig. 4), and in the case of Pb and Zn the coefficients were 0.4. Therefore, outdoor dust influences indoor dust metal concentrations to some extent but is not the main source of metals inside homes, which is consistent with the O/I result. On the other hand, the outdoor concentrations of Cu, Pb, and Zn had correlation coefficients higher than 0.8; the same happened inside, which suggests that these metals come from the same sources outdoors and indoors, although they may be different between both environments, for example, outdoors they can come from the vehicle fleet and industries, while indoors they can come from cement and paintings.

Other strong positive correlations occurred between Fe and Mn (*r* > 0.9) outdoors and indoors, as well as between Ti, Fe, and Mn ( *r* > 0.6), and between Sr and Y (*r* = 1). However, no relationship was found between the outdoor and indoor concentrations of these elements. This suggests that these metals tend to have sources in common in each environment (outdoors and indoors) but independently so that the sources from outside the houses differ from those inside.

### Site evaluation

In general, it can be said that most indoor dust is contaminated with heavy metals, but very few dust samples are not (contamination factor less than 1). Considering the average values of heavy metals, it is observed that contamination ranges from moderate to considerable, with isolated cases of high contamination. Pb, Cu, and Zn are the heavy metals with the highest contamination factor values; they are the ones that should be monitored.

**Fig. 5:**
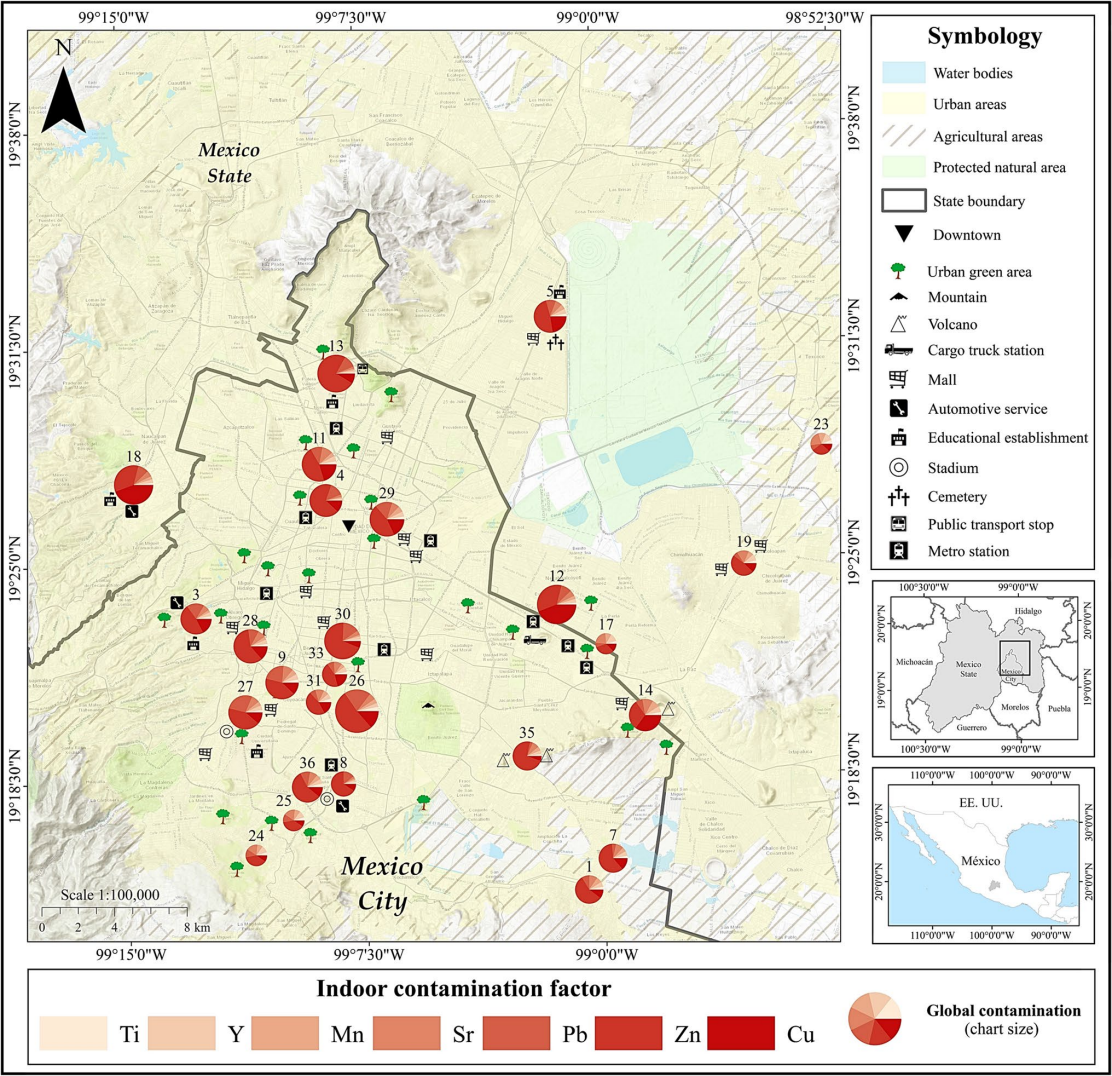
Contamination factor and *PLI* by sampling sites indoors in Mexico City

The population that should be alerted is the one that inhabits the houses of sites 4, 5, 11, 12, 13, 14, 17, 26, 27, 28, 29, and 30 (Fig. 5). To the north (site 13) and the northeast (site 5), Zn was the heavy metal with the highest contamination factor value, followed by Cu and Pb. Both points are located in the area with the most significant industrial activity, such as medium-sized industries manufacturing food and beverages, leather and textiles, agro-industrial, electronic, and electrical products; logistics companies (distribution of medicines, supermarkets, supplies and materials for home repairs and electronics); and high population density. At the center of Mexico City, sites 11, 4, and 29, the metals with the highest contamination factors follow the sequence Zn > Pb = Cu. The zone is mainly high-traffic vehicular, commercial, and services. To the south of Mexico City, sites 26, 27, 28, and 30, the metals with the highest contamination factors follow the Zn > Pb > Cu sequence. The area is not densely populated but has high vehicular traffic because it is a service and residential area. To the southeast, we find site 35 between two extinct volcanoes, where the Zn contamination factor is the highest value (Fig. 5).

To the east of Mexico City, sites 12, 17, and 14 have the metals with the highest contamination factors: Cu, Zn, and Pb. These sites are located on the border with the state of Mexico; the area has a high population density and high road traffic and is very close to Lake Texcoco, a sizeable, deforested area where dust storms frequently occur (Fig. 5). At site 18, located west of the city, Pb was the heavy metal that presented the highest value of the contamination factor, above Zn and Cu (Fig. 5).

**Fig. 6:**
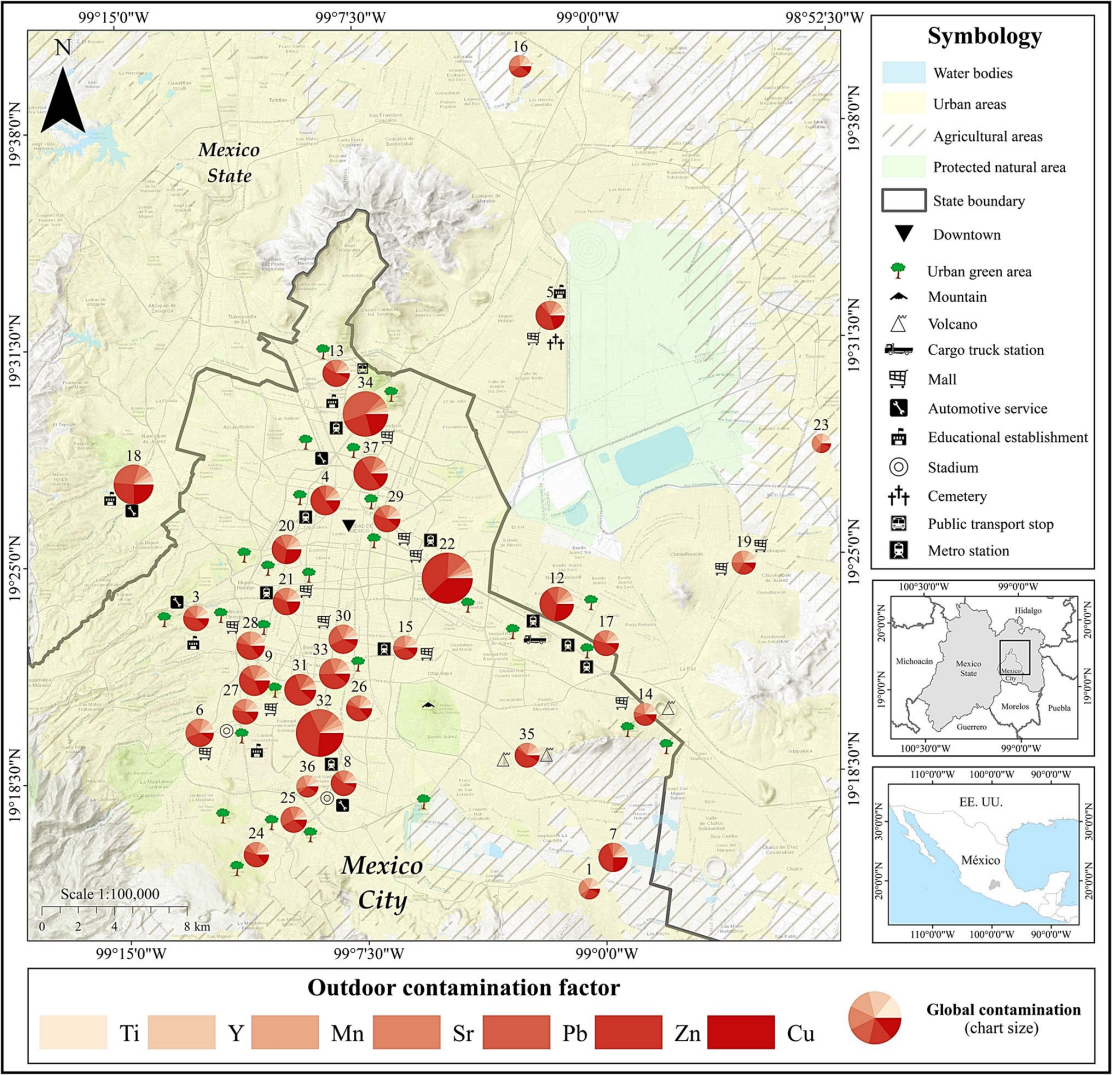
Contamination factor and *PLI* by sampling sites outdoors in Mexico City

The sites with high values of the contamination factor outdoors are to the north 13, 5, 34, and 16; center 37 and 4; south 30, 31, 32, and 33; to the east 12 and 22; and the west 9, 28, and 6. At site 16 to the north of the city, Pb had a higher contamination factor value almost the same as Zn, perhaps because it is located in the industrial area. In this case, the government of Mexico City is responsible for cleaning the streets (Fig. 6).

The results indicate that contamination is higher indoor than outdoor in all cases and for all metals measured.

### Human health risk assessment

**Fig. 7:**
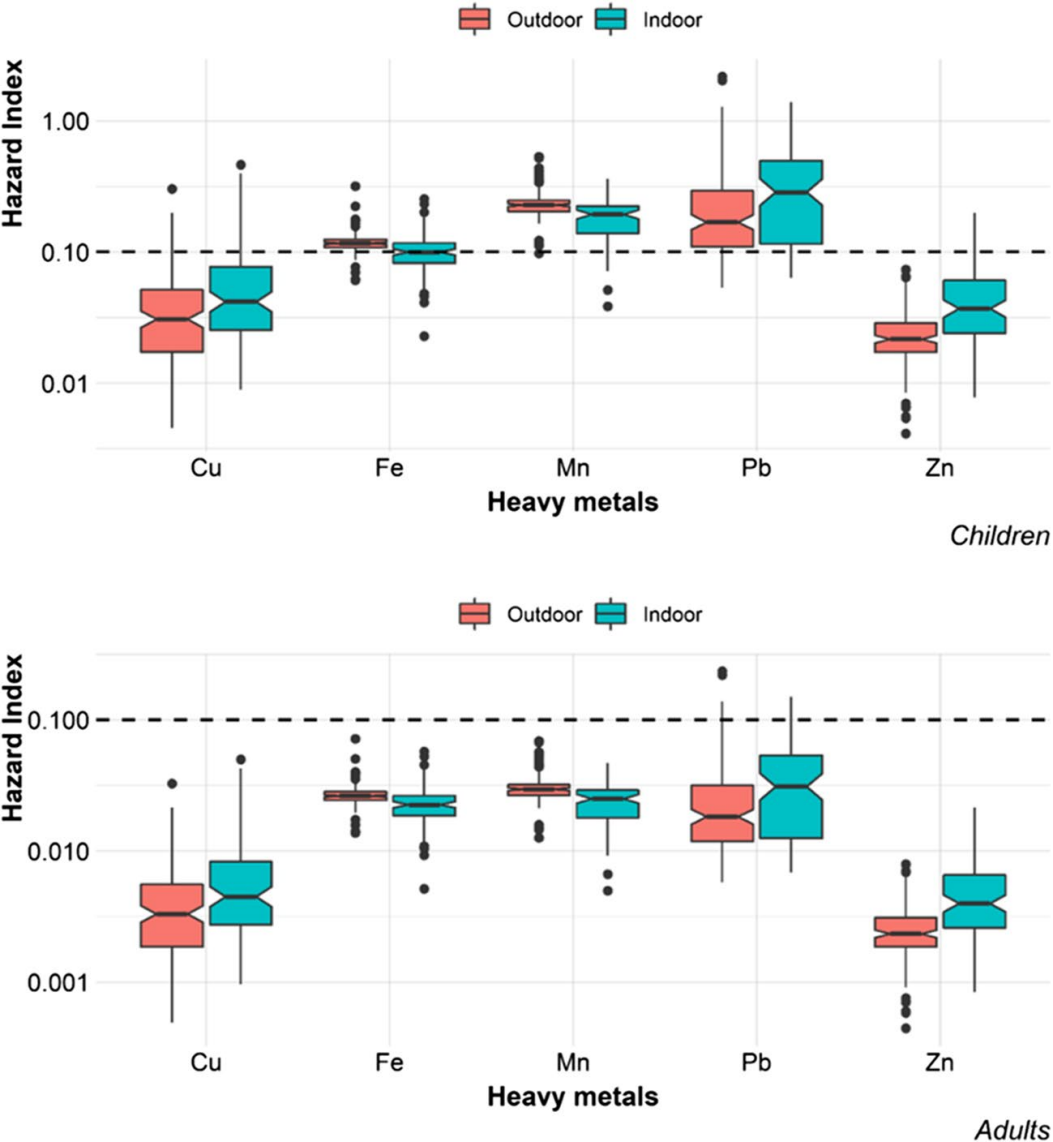
Hazard indexes for Pb, Mn, and Fe, in some cases, were higher than 0.1 (dotted line); therefore, ailments can be triggered (Jadoon et al. 2018) in children’s health (top image). For adults, hazard indexes for all heavy metals (Cu, Fe, Mn, Pb, and Zn) were lower than 0.1 (bottom image)

The human health risk was not detected, as the hazard index was lower than 1. However, ailments can be developed due to exposure to Pb, Mn, and Fe in children (HI > 0.1). A higher risk due to Pb exposition was found indoors (Fig. 7). The health risk from exposure to Fe and Mn was higher outdoors. Cu and Zn were the metals that had the lowest risk to the health of the Mexico City population. The main route of exposure for all metals was ingestion; only in the case of Fe, both ingestion and inhalation were the most important routes of exposure. A carcinogenic risk was not identified, as the maximum *RI* for Pb was still below the tolerable risk range *(RI*_max_ = 7.536E − 09).

Pb is one of the metals of greatest interest in Mexican cities since it is found in high concentrations in outdoor dust and represents an ecological and human health risk (Aguilera et al. 2022). In addition, the levels of lead in the blood of Mexican children exceed the limit established by the Centers for Disease Control and Prevention of the USA (Caravanos et al. 2014). Therefore, it is extremely important to reduce the concentrations of this metal in the urban environment, especially inside homes.

Some limitations of this human health risk assessment should be mentioned. Firstly, this assessment was carried out with total concentrations of heavy metals, while only some concentration is bio-available to the human body through ingestion or dermal contact; for inhalation it depends on the particle size. Secondly, the exposure factors were taken from reference populations, so the actual exposure of each resident varies depending on their exposure times, their body weight, and their predispositions. Third, other sources of exposure, such as water and food, were not taken into account in the analysis, so the risk may also be underestimated in that sense. The results reported here are for reference only.

In recent studies, Mn has been identified as one of the metals that represent a greater risk to the population’s health, especially in indoor environments (Sajedi Sabegh et al. 2022; Santoyo-Martínez et al. 2022; Zhou et al. 2022). This finding has demonstrated the importance of including metals considered major elements in studies of environmental contamination and human health risk. Even though the human body has mechanisms to regulate the concentrations of metals such as Mn and Fe, in excess, they can represent a problem.

### Minerals in the urban dust

As for the most abundant minerals in the urban dust indoor and outdoor of Mexico City, we find quartz, calcite, and anorthite. In the urban dust samples from indoor and close to the former Texcoco lake, we also found Tosudite [Na_0.5_(Al,Mg)_6_((Si,Al)_8_O_18_)(OH)_12_·5H_2_O], Chabazite [Na2Ca(Si8Al4)O24·12H_2_O], Zussmanite [K(Fe^2+^,Mg,Mn)_13_[AlSi_17_O_42_](OH)_14_], Dawsonite (NaAlCO_3_(OH)_2_), and Ferro-actinolite (Ca_2_(Mg_4.5 − 2.5_Fe2 + _0.5 − 2.5_)Si_8_O_22_(OH)_2_). However, these minerals are not found in the urban dust north of the city.

In the urban dust outdoors, we find that the main minerals are Quartz (SiO_2_), Calcite (CaCO_3_), and Anorthite (CaAl_2_Si_2_O_8_), but there is also Orthoclase (KAlSi_3_O_8_), Thermonatrite (Na_2_CO_3_·H_2_O), and Ferro-actinolite.

The results of the XRD analysis allowed the identification of urban dust minerals, mainly those of natural origin, of which we found two sources, the dust of sedimentary origin from the former lake of Texcoco and the dust that originates from the weathering of the igneous rocks from the volcanoes of the city and its surroundings.

We thought we could find mineral differences in indoor and outdoor dust because magnetic minerals such as maghemite and magnetite come from combustion gases (industry and automobiles). However, they are non-dominant minerals in the mineral matrix of the dust. Other studies have reported the presence of minerals of anthropic origin identified with magnetic parameters, such as magnetic susceptibility and saturation isothermal remnant magnetization (Cejudo et al. 2022; Cejudo Ruiz et al. 2022), and have documented their toxicity in brain, lung, and liver (Hammond et al. 2022).

It is necessary to study the particles of anthropic origin more detailed with more samples, to identify the minerals’ shapes, sizes, and possible toxicity.

## Conclusions

The highest contamination factors were those for Pb, Zn, and Cu, followed by Sr and Mn, using both local and global background values. The contamination was higher when the global background value was used since there is naturally high contamination in Mexico City.

The Pb, Zn, and Cu contamination was significantly greater indoors, while higher Mn, Sr, and Fe were detected outdoors. We consider three possible explanations for the higher Cu, Pb, and Zn content indoors, which are not exclusive but complementary: (1) there are important sources of these metals indoors; (2) there is a greater accumulation of metals indoors, especially if ventilation is poor; and (3) due to the decrease in outdoor activities because of the COVID-19 confinement, outdoor emissions decreased and those indoors ones increased.

According to the outdoor/indoor ratios, the main sources of Cu, Pb, Zn, and Ca are indoors, with some influence from outdoor dust because moderate correlation coefficients were found between outdoor and indoor concentrations. On the contrary, the main sources of Fe, Mn, Sr, Y, and Ti are outdoors, and they are different from the indoor ones.

The human health risk was not detected, as the hazard index was lower than 1. However, ailments can be developed due to exposure to Pb, Mn, and Fe, in children (HI > 0.1). A higher risk due to Pb exposition was found indoors.

In Mexico City, indoor environments were more contaminated by heavy metals and represented a higher risk to human health than outdoors during the COVID-19 isolation.

## Data Availability

The raw data supporting the conclusions of this article will be made available by the authors, without undue reservation.
